# Medical News and Miscellany

**Published:** 1892-09

**Authors:** 


### Medical News and Miscellany.

---

"The Happy Medium."—We are glad to know it is "happy;" it certainly is "medium."

---

Dr. W. P. Perkins has removed from El Paso, Texas, to Bastrop, Morehouse Parish, La.

---

Dr. D. R. Wallace has returned to Waco after ten weeks sojourn and travel in the great Northwest, including Alaska.

---

The office of Drs. R. S. and O. L. Williams, at Dallas, was burned recently, and they lost all their books and instruments.

---

Dr. C. M. Harrison, formerly health officer at Tampico, Mexico, and who left there on account of hemorrhages of the lungs and went to Silao, away up 6000 feet higher, the JOURNAL is gratified to learn, has entirely recovered his health. He has removed now to Panneo, Mexico, near which place he own a valuable ranch.

**Died.**—In Ridgetown, Ontario, Canada, Thursday, August 25, 1892, Dr. T. W. Schlenker, aged 24 years and 11 months. Dr. Schlenker spent last winter in Austin, in the hope of recovering his lost health. He was a victim of phthisis. Dr. Schlenker was a bright young physician, and had he lived he would have made his mark in the world.

---

The firm of Drs. Holmes & Davis, of Rome, Georgia, has been dissolved, Dr. Davis withdrawing. He goes back to Birmingham, Alabama, and Dr. J. B. S. Holmes continues in charge of his splendid infirmary, as before. Attention is called to his advertisement in this issue. As a gynecologist, Dr. Holmes has few superiors, and the climate and surroundings of Rome are valuable accessories in the treatment of the class of patients who seek his services.

---

At the district court, on Tuesday, May 31, G. H. Raymond was fined £20, with five guineas cost, for having falsely described himself on the 13th April as a "botanical physician, oculist and aurist," whilst the evidence showed that he was not legally registered by the Medical Board. On the defendant pleading that the fine was excessive and would compel him to go to goal the bench reduced the fine by £5.—*Pharmaceutical Journal of Australasia.*

---

**Dr. F. E. Yoakum**, formerly of Greenville, Texas, and more recently of Shreveport, Louisana, a member of the Texas State Medical Association, and a particular favorite with the Texas profession, has been elected to the chair of Materia Medica and Therapeutics in the Gross Medical College, at Denver. The JOURNAL extends congratulations to the doctor that notwithstanding his great modesty his merits have been discovered and appreciated; and to the Faculty of Gross Medical College on the acquisition of so able, genial and popular a confrere. The doctor will be popular with the students, and we should not be surprised if he prove a strong counter-attraction to our owns Galveston school, with its popular Faculty.

---

**Alice Mitchell's Case.**—The *Mississipi Valley Medical Monthly*, Memphis, Tennessee, gives, in the August number, a complete detailed report of the trial of Alice Mitchell to determine whether or not she is insane, together with a full report of the expert testimony given by those distinguished specialists, Sim, Callendar, Ingram, Fletcher, Hammond and Sale. It is intensely interesting reading, and the profession are under obligations to the *Medical Monthly* for this, the only full report, we believe, yet published. It is a source of much satisfaction to note the entire absence from the case of any disgusting features; "sexual perversion" appears to have had nothing to do with the case, and no physical examination of the unfortunate girl was made, or necessary. It was clearly a case of Platonic love,—in an emotional young woman who had inherited the insane predisposition, and in whom insanity in the form of paranoia had developed through jealousy, acting as an exciting cause.

---

Married—Aug. 18th ult., at Sulphur Springs, Texas, Dr. E. P. Becton to Mrs. Josephine Morris, both of that city.

The JOURNAL extends its cordial congratulations to the worthy and gifted disciple of Galen and Esculapius. He deserves a good wife, and being a man of sound and mature judgment, we are sure he has selected such. David of old took two virgins to "warm him up." Dr. Becton is always "warmed" up and can impart warmth. Its a blessing to hear him warm up in a speech. The Doctor has raised a family and has one son a doctor and one son-in-law in the same line, yet he is not an old man by any means. Age is not counted by years; he is yet young in heart and in sentiment and in full vigor of mental strength. Oliver Wendell Holmes boasted that he was "80 years young." So with our friend Becton, he is "—— years young."

